# Supplementary material for: Are carbon emissions trading and green financial instruments synergistic? -Comprehensive quantitative research based on content analysis
Source: PLoS One. 2024 Mar 7;19(3):e0298601. doi: 10.1371/journal.pone.0298601 (PMC10919601; doi:10.1371/journal.pone.0298601)
Supplement: S1 Data — (ZIP) [file pone.0298601.s001.zip › Data/地级市Z_F工作报告.zip!/╔┬╬≈/░▓┐╡╩╨/└┤╫╘╣1⁄2╓┌║┼í╛┬φ┐╦╩2╛▌═°í┐_2014.docx]

各位代表：

现在，我代表市人民政府向大会作工作报告，请予审议，并请政协委员和列席会议的同志提出意见。

一、2013年工作回顾

2013年，在市委的坚强领导和市人大、市政协的监督支持下，我们紧紧依靠全市人民，围绕循环发展、富民强市主题和转变经济发展方式主线，突出项目、产业、城镇化三大重点，统筹改革、民生、增长极培育三项工作，经济发展稳中有进，社会和谐稳定，完成了市三届人大三次会议确定的各项目标任务。

(一)经济平稳增长，质量效益提升。完成生产总值604.6亿元，增长13.4%，高出全省2.4个百分点；财政总收入58.6亿元，增长13.2%，其中一般预算收入25.3亿元，增长17%；城乡居民收入分别达到2.25万元和6624元，增长11%和13.9%。工业化率41%，非公有制经济增加值占GDP比重50.9%。节能减排任务完成，单位生产总值能耗下降3.3%。

(二)循环产业壮大，发展动力增强。新型工业化发展加快，完成规模以上工业增加值231亿元，增长24.8%(全省第二)，新增规模工业企业62户、产值过亿元企业74户，工业短板状况正在改变。现代农业持续增长，粮食产量86万吨、油料产量14.4万吨，生猪出栏310万头，发展设施蔬菜16万亩，富硒茶叶、魔芋、水产养殖、林下种养等规模效益增长，新建特色经济林35万亩，绿化造林65万亩；培育市级以上农业龙头企业78户，职业农民培育工作得到农业部推广，富硒产品研发、院地合作成效明显。生态旅游长足发展，瀛湖跻身全省十大重点文化基地项目之列，重点景区建设和乡村旅游发展加快。三产服务业健康发展，新增限额以上商贸企业58户，社会消费品零售总额增长13.4%。

(三)项目建设有力，投资拉动明显。完成全社会固定资产投资482.6亿元，增长26.9%，民间投资占投资总量的60%。安康火电厂、白河水电站、旬阳烟厂技改、金属镍产业园等重大产业项目取得实质进展。上海复星集团、海尔物流落户安康，西北国际天贸物流城项目启动实施，帝奥电梯、北医大技改、正森富硒饮品等40个工业项目竣工投产。基础项目建设加快，十天高速白河至十堰段、西康铁路复线通车，安康富强机场进场路开工。西成客专宁陕段、安平高速、集中连片扶贫县乡公路建设加快推进。城区西郊110千伏输变电站建成。汉江综合整治及城区东坝、5县城防洪工程进展顺利，江北和8个县城污水处理厂建成运行。完成42个重点减排、35个污染治理项目。瀛湖列入全国15个重点湖泊生态环境保护试点项目；“气化安康”进度加快，2万户居民用上天然气。

(四)统筹城乡发展，城镇化步伐加快。以中心城市为龙头，以移民搬迁和农民进城为抓手，城镇综合承载力增强，城镇化率达41%。按照民生城建的思路，加大中心城市重点市政项目建设力度，积极破解商业步行街、南环东路、西郊公园等项目征地拆迁、环境保障难题，投资18.2亿元实施十大市政重点项目，南环东路、兴安公园、西城阁、金州广场提升改造、两座人行天桥建成，城东大桥一期、江滩公园、汉江龙舟文化园、兴康门、汉江一桥交通组织等工程加快建设。创卫创园通过国家暗访和实地考察，五县一镇进入国家卫生县镇行列。县城、重点镇和新型社区建设步伐加快。避灾扶贫搬迁建房2.2万户8.36万人，集中安置率85%；农村居民进城落户9万人。

(五)实施园区突破，承载能力增强。高新区、恒口示范区、瀛湖生态旅游区和县域工业集中区、现代农业园区 “三区两园”突出基础建设和招商引资，综合承载力提升；打破行政区划限制，创新规划、建设、管理和利益分配等工作机制，“飞地经济”园区加快建设。高新区完成投资35亿元，道路、公园、广场、学校及一批文体设施建成，19个产业项目竣工。恒口示范区完成基础建设投资4.5亿元，省级重点示范镇建设加快。瀛湖生态旅游区按照“湖城一体”的思路，深化与陕文投合作，启动规划编制，创新经营管理体制，加快基础设施建设。县域工业集中区完成投资48亿元，新建标准化厂房15万平方米。现代农业园区以壮大龙头企业为重点，完成投资46亿元，实现产值52亿元。

(六)重点改革深化，对外开放扩大。加快推动全国发展改革试点、国家主体功能区创新示范工作，创新县域工业集中区管理机制，中心城市管理、公共交通以及公安、国土、教育、卫生和食品药品、文化体制、事业单位分类改革加快推进。金融创新步伐加快，设立金融仓储公司、旬阳民生金融创新示范县以及宁陕集体林权、平利土地流转经营权和农房抵押贷款等工作积极开展，2家农村信用社改制为农村商业银行，市级融资平台市场化融资能力增强，金融支持发展的力度加大。探索实践民间资本进入基础设施、城镇建设、社会事业领域。招商引资到位资金267亿元，引进劳动密集型企业64户。

(七)民生持续改善，社会建设加强。完成民生工程投资95亿元。新增城镇就业1.75万人，农村劳动力转移就业68万人。财政专项扶贫投入21.6亿元，实现10.6万贫困人口脱贫。开工保障性住房3.75万套，建成2.78万套，累计分配入住5万套。完成教育项目投资8.5亿元，加大县城中学建设力度，新建和改扩建幼儿园70所，学前教育和高中入学率分别达93%和92.8%；中心城区教育资源整合加快推进，按“五校合一”推动以安康职业技术学院为主体的北迁。加强公共卫生和医疗服务体系建设，创新新农合和公共卫生服务管理、卫生人才培养机制。人口和计生工作加强，低生育水平保持稳定。城乡居民社会养老保险、城镇基本医疗保险、新农合实现全覆盖。安康剧院、博物馆、儿童医院、妇幼保健院、精神病专科医院等建设顺利。文化事业和产业发展加快，文化产业增加值增长27.8%。汉江水质保护、节能减排和环境治理工作加强，生态示范创建工作走在全省前列。创新社会治理，9个县实现信访“三无”目标。实施汉滨公安管理体制调整，完善社会治安防控体系，社会治安满意率居全省前列。“六五”普法深入开展，人民调解工作加强，依法行政示范市创建工作深入推进。安全生产“四项指标”下降，防灾减灾和胡蜂防治有力有效，妥善解决社会关注的热点问题，为改革发展营造了良好环境。

(八)自身建设加强，行政效能提高。我们把加强自身建设作为头等大事，严格执行中央八项规定，推进法治、诚信、责任、服务、廉洁政府建设，完善政府工作规则，自觉接受市人大及其常委会的法律监督和市政协的民主监督，主动向人大代表、政协委员、老干部通报经济社会发展情况。办理人大代表建议190件、政协提案309件。加大信息公开力度，拓宽联系群众渠道，邀请66名人大代表、政协委员、群众代表和41名企业代表旁听市政府常务会和全体会，125名公民代表参加“走进市政府”活动。建立重点工作公开承诺、督查通报机制。扎实开展规范公务用车、办公用房等专项治理。69个市直单位实行预决算公开试点，“三公”经费降低17%。深入开展狠刹“五股歪风”和优化投资环境专项治理，完成土地例行督察整改。全面推进依法行政，强化行政监察和审计监督，廉政建设加强。其他各项工作都取得了新成绩。

2013年各条战线创先争优、奋发有为，成绩来之不易，是市委坚强领导，市人大、市政协和社会各界大力支持，全市人民奋力拼搏的结果。我代表市人民政府，向奋战在各行各业、各条战线的广大干部群众，向人大代表、政协委员和各民主党派、工商联、人民团体、民族宗教等各界人士，向离退休老干部、驻安人民解放军、武警和消防官兵、政法干警，向关心支持安康发展的社会各界，致以崇高的敬意和衷心的感谢!

在肯定成绩的同时，必须正视经济社会发展中存在的困难和问题：发展不足，结构不优，特别是工业短板还没有破解；基础设施保障能力不强，特别是交通互通互联水平低，抵御灾害能力弱；资源环境约束趋紧，生态建设和环境保护任务艰巨；发展环境不优，对外开放水平不高，政府职能转变还不完全到位，公共服务能力水平低，一些地方和部门存在着不负责、不敢担当、落实不力的问题。要直面问题，不以事艰而不为，不以任重而畏惧，采取有力措施加以解决，让人民群众看到实实在在的变化和成效。

二、2014年目标任务和措施

2014年经济社会发展面临难得的历史机遇。中央、省委、省政府和市委对改革发展作出了全面部署，特别是春节前李克强总理亲临安康视察，指出，“在汉江两岸拔起了安康这样一座现代化城市，出乎我的预料”，“在秦巴山区腹地嵌上了一颗明珠”，“安康虽然地处秦巴山区腹地，反过来讲，也有区位优势，有可能形成、也正在形成这一集中连片区域的交通枢纽”，“我这次到安康，汉江两岸灯火辉煌，而且雄心勃勃要在秦巴山区腹地开花，敢于向四周开放、敢于竞争”，希望安康“在秦巴山区中心开花，开出大花、结出硕果”，提出“国家将给予安康必要的支持和更多的倾斜”。李克强总理的重要讲话，体现了党中央、国务院对安康发展的重视和关怀，指明了安康今后发展的方向，进一步坚定了我们建设美丽富裕新安康的信心和决心。我们要以李克强总理视察为动力做好今年各项工作，以开展党的群众路线教育实践活动为契机推动改革发展，以贯彻落实中央八项规定的坚定性促进政府作风转变，推动安康的事业不断前进。

工作的总体要求是：认真贯彻落实十八届三中全会、省委十二届四次全会和市委三届五次全会精神，突出循环发展、富民强市主题，紧扣转变经济发展方式主线，以提高发展质量和效益为中心，以促进社会公平正义、增进人民福祉为出发点和落脚点，把改革创新贯穿各领域各环节，构建循环产业体系，改善基础设施条件，加快新型城镇化步伐，加强生态文明建设，加大民生改善力度，确保经济持续健康发展和社会和谐稳定。

发展的预期目标是：生产总值增长12.5%；全社会固定资产投资增长24%；财政总收入和地方一般预算收入分别增长10%和13%；城乡居民人均收入分别增长11%和13%。人口自然增长率控制在5‰以内，完成省定主要污染物减排和节能降耗任务，汉江出境断面水质保持国家Ⅱ类标准。

围绕上述目标，重点做好八个方面工作：

(一)狠抓项目投资，保持经济平稳较快增长

优化投资结构，提高投资效益，激发社会投资活力，全年投资增长24%，重点加大优势产业、基础设施和农业、民生、生态投资力度。实施100个市级重点项目，完成年度投资230亿元。

实施重大产业项目攻坚。全力推动安康火电厂、白河水电站、旬阳烟厂技改、金属镍产业园等财政翻身项目开工建设。突出抓好旬阳电站、荣氏食品、皓翔塑钢等60个产业项目，完成年度投资66亿元。围绕建设区域物流中心，开工西北国际天贸物流城，推进高客站、恒口500万吨铁路货场和朝阳产业城等项目建设。

加快以交通为重点的基础建设。加快安平高速、安康富强机场、西成客专(宁陕段)、阳安复线、石紫岚公路、平镇二级路、中心城市及县城国道改线(中心城区北环线，白河、汉阴、石泉国道过境线)、十天高速旬阳连接线、五座汉江大桥、安康至白河高等级航道、安康中心港以及连片扶贫县乡公路建设，完成旬小、安旬、旬白、宁两二级公路前期工作。加强农村电网改造，实施旬阳330KV、城区东郊110KV输变电工程。

抓好生态、农业和水利等项目。加大瀛湖湖泊生态环境保护、富硒产品研发中心、引汉济渭、洞河水库和现代农业产业园项目建设力度。加强汉江综合整治，抓好城区东坝、沿江县城、重点镇和月河、黄洋河等防洪工程和病险水库治理。推进旬阳、恒口、建民、高新区污水处理厂和沿江河集镇、集中安置社区的水利、污水和垃圾处理设施建设。

谋划关乎长远的重大项目。围绕建设区域交通枢纽和重点产业发展，强力推进平镇高速、安岚高速、石泉至宁陕高速连接线，西渝、西武高铁和安张衡铁路，月河快速干线、汉江高等级航道、月河补水等重大基础项目，力争开通安康至西安的动车；下力气抓好月河川道城镇带、石紫岚沿江产业经济带和石煤综合利用、富硒特色农业等产业项目；围绕生态宜居谋划旅游度假、健康服务、养老服务、保健养生等产业；围绕区域物流中心推进综合物流园区、物流信息网络平台、农产品冷链物流、电子商务和配送体系等建设。不断扩大民间投资，使民间投资成为固定资产投资的主导力量。

(二)做大做强产业，构建循环产业体系

把生态友好型产业发展作为产业结构优化升级的重要内容，坚持做大产业规模与优化结构并举，延伸产业链条，实施品牌战略，提升产业发展层次、质量和效益。

加快新型工业化进程。提升清洁能源、富硒食品等支柱工业，培育壮大装备制造、新型材料、生物医药等新兴工业，大力发展中小微企业和劳动密集型企业，提高工业运行质量和效益，培育规模以上企业50户，规模以上工业增加值增长22%。装备制造业重点支持神达、俊达、宝通、亨通、南水汽配等企业扩大产能，提高市场占有率，抓好配套产品研发和引进，建设汽车制造配套基地。新型材料优先发展非金属和建筑新材料，把龙头企业培育壮大和延伸产业链作为主攻方向，支持旬阳中科纳米、岚皋兴瑞达材料、汉阴兴科建材、平面发热材料、汉滨国群非金属矿等企业做大做强。生物医药以药源基地建设、新产品研发和深化对外合作为重点，提升正大、北医大、镇坪制药厂等医药企业竞争力，支持医药中间体和中药饮片企业发展。电子信息支持众辰钟表文化产业园、博瑞特半导体、凯翼通等企业提高效益。富硒食品以研发中心为重点，抓好精深加工，培育龙头企业，做大真硒水、矿泉水、富硒茶饮果饮及烟草等产业，建设全国富硒食品基地，打造中国硒谷地域品牌。

大力发展现代农业。加大“三农”投入，落实好各项强农惠农富农政策。严守耕地红线，稳定粮食生产，保持总产80万吨以上，建设高标准基本农田10万亩。抓好蔬菜生产供应，强化“菜篮子”市县区长负责制。以重点产业园区为载体、市场为导向、企业为主体，加快实施国家级特色(富硒)高效农业院地合作示范区规划，打造富硒特色高效农业产业链。做大做强富硒茶产业，重点抓好4个主产县区和40个基地镇、100 个专业村建设。大力发展山林经济，提升林下种养经营水平；加快以阳晨牧业为重点的生猪产业、以嘉晟、忠诚园区为重点的蔬菜产业发展，扩大魔芋、绞股蓝、中药材等产业规模，提高蚕桑、烤烟、特色种养效益。充分利用水资源优势，科学规划和推进涉水产业发展。加大重点龙头企业培育力度，推进农业生产经营方式创新，扶持农民专业合作组织，培育新型职业农民，加强基层农技推广体系建设，保障农产品质量安全。

突出山水休闲旅游。加快“一山一湖一城”核心景区建设和特色旅游板块发展。重点抓瀛湖旅游产业基地、中心城区一江两岸核心景区建设和县区精品培育，把汉滨双龙、岚皋南宫山、石泉后柳水乡、宁陕“山水秦岭”、平利“美丽乡村”等打造成精品景区，加快旬阳蜀河、汉阴双河口和漩涡、石泉古城、白河桥儿沟等文化旅游名镇(街区)建设。带动现代服务业发展，扩大信贷刺激消费领域，大力培育文化、休闲、健康、养老、电子信息等消费业态，支持房地产市场健康发展，推动农村居民住房、文化和家电消费，加快农产品流通体系建设。加快文化产业发展，培育壮大新型文化业态，推动网络视听、动漫游戏、数字出版、时尚创意等产业发展。培育限额以上商贸流通企业40户，社会消费品零售总额增长13%。

壮大“一县一产业”。根据主体功能区规划和资源禀赋，发挥县区资源、文化、交通、人才等比较优势，明确定位，突出重点，在做大做精做特上着力用功，提升竞争力，壮大特色产业。加大支持力度，发挥汉滨区的龙头带动作用，提高旬阳对全市发展的贡献率；发挥比较优势，突出抓好汉阴富硒食品，石泉优美县城和特色旅游，紫阳富硒茶饮，平利“美丽乡村”，白河汽车装配，岚皋、宁陕、镇坪生态旅游和特色种养发展，发挥举旗抓纲的作用，带动县域经济发展。

(三)推进园区集聚集约发展，发挥示范引领作用

坚持统一规划、分步实施，完善基础条件，增强综合承载能力，把“三区两园”和“飞地经济园区”打造成循环产业聚集区、全市经济发展的增长极。

高新区以建设现代城市新区、高新产业聚集区和创新示范区为重点，打造产城结合、创新型的生态新城。围绕城市重心北移，加快路网配套，完善路、水、电、气、绿地、通讯等基础设施，加快教育、医疗、文化、体育等社会事业发展，推进市场、商贸、餐饮等要素完善；加快新材料、食品生物、装备制造、电子信息、金融服务、健康服务等新兴产业培育；围绕创建国家高新技术产业开发区目标，广泛开展技术创新合作，推进科技资源集聚，增强自主创新能力，加强园区融资、企业孵化、创业支持、技术创新、人才引进培育等平台建设，推动投融资体制、管理模式和政策环境创新。瀛湖生态旅游区重点抓规划编制和以基础设施为主的项目策划、争取，推进与陕文投合作取得实质性进展，加快整体开发。严格规划管理和资源保护，实施瀛湖生态环境保护项目，按湖泊承载能力科学发展水产养殖；结合移民搬迁和产业发展，提升瀛湖、流水及旅游村的规划建设水平。恒口示范区以建设市域副中心为目标，以省级重点镇建设为契机，完善区域性总规、建设详规和土地利用、产业发展规划，按照统筹城乡、产业支撑的要求，加快推进避灾扶贫搬迁跨区域安置，加大基础改善、产业发展、商贸繁荣和环境美化项目实施力度。县域工业集中区以旬阳生态、汉滨五里、石泉古堰、汉阴月河、平利陈家坝、白河红石河、紫阳硒谷等园区为重点，抓好道路、管网、污水垃圾处理设施及标准化厂房建设。加快“飞地经济”园区建设，进一步完善政策支撑和保障体系，创新“飞出地”和“飞入地”协作机制，突出抓好项目和企业落地，做大做强入园企业。现代农业园区完善道路、电力、灌溉等设施，实施“十园示范百园提升”工程，推进标准化生产和无公害农产品、绿色食品、有机农产品发展；吸引工商资本进入，创新土地经营权流转模式，壮大龙头企业。坚持园区集聚集约发展。搞好产业规划，提高项目入园门槛，在产业、技术、环保、用地等方面从严要求，提高投资强度和供地门槛，清理以各种名义的“圈地”行为，防止粗放经营或产业“同质化”，重点引进节能环保、带动和成长性强的企业，推进产业链延伸，形成产业集群。

(四)打造“秦巴明珠”，加快新型城镇化步伐

“秦巴明珠”既是李克强总理对安康发展的肯定和生态城市的赞美，更是对我们的殷切期望。“明珠”还不名副其实，“璀璨”还需倍加努力。要坚持以人为本、产城融合，科学规划城镇规模和布局，强力推进中心城市建设，加快月河川道城镇带和县城、集镇、新型农村社区建设。

建设秦巴腹地璀璨明珠。以建设区域中心、生态宜居城市为目标，突出核心，提升江南，重心北移，努力把中心城市建设成为秦巴腹地的一颗璀璨明珠。核心围绕“湖城一体”，把瀛湖与中心城市建设有机结合，加快推进城东大桥、江北商业步行街、滨江1.2.3号地、汉调二黄广场二期、江滩公园等重点工程，实施“一江两岸”环线游项目，规划建设东关旅游特色小吃街区。江南重点抓严控、抓安居、破难题、促提升，控制开发强度，完善公共设施，实施供销车队和安运司搬迁，推进西郊公园、殡仪馆搬迁以及城东棚户区改造、南环东路、高井路和东坝片区基础设施等重点项目建设。江北坚持规划引领、高新引擎、基础先行，力促江北火车站片区、月河生态城跟进高新区建设步伐。围绕富强机场规划建设空港经济区，依托西北国际天贸物流城建设现代物流园区。江北火车站片区重点建设北环线和宗教文化体验区，规划建设大型工程机械交易市场，推进张岭、安铁、黄沟片区棚户区改造。月河生态城坚持基础先行，开工建设长春路、新罗路、秦岭大道。在城市建设中立足长远，树立精品意识，从城市的整体规划建设到每个单体建筑，都精心策划、设计和施工，让建筑成为作品，彰显文化内涵，把安康打造成一个宜居宜业宜游的生态之城。

做美县城、重点镇和新型社区。坚持因地制宜、突出特色，走集约节约和内涵深化的路子，按照建设生态化、管理精细化的要求，做优、做精、做美县城，突显山、水、绿的特色，延续城市历史文脉，完善公共服务功能，防止“摊大饼”式的盲目扩张，使县城显山露水、山水相依、生态宜人，增强群众幸福感。按照规模适度、生活便利、群众安逸的要求，加快重点镇建设，完善公共设施，每个县至少抓一个重点镇，建成县域副中心。按照服务周到、便利生活、居民满意的要求，加强社区服务设施建设，抓好社区医疗计生、文体教育、环境卫生等公共服务。加快发展就业容量大的三产服务业和劳动密集型企业，大力发展特色产业，吸引农村居民就地城镇化。

完善城市基础设施和要素。抓住国家加强中西部城市基础设施建设的机遇，加大市政基础项目建设力度。完善城市道路网络系统，加强交通拥堵路段、节点的改造。下定决心、排除万难，把城市公共交通发展好、管理好，重点优化公交网布局，扩大覆盖半径，建设调度中心、停车场、保养场、首末站以及停靠站，形成客运“零换乘”交通体系；规划建设城市步行、自行车“绿道”和停车场、人行过街、道路照明设施，实施城区背街小巷改造。加强城市防洪(涝)、供水、污水、雨水、天然气、电力、通信等各类地下管网的建设改造，推进地下综合管廊建设；加强城市电网建设，保障城区和园区电力供应，推进重点区域架空线缆落地；加快城镇教育、医疗卫生、文化、体育、避险、养老、救助、社区服务、社会管理等公共服务设施，以及各类专业市场、物流园区、物流配送基地及其仓储设施建设。实施增绿扩绿工程，加大城镇公园、街头游园、湖池湿地、绿道绿廊等规划建设力度。

提高城镇建设管理水平。把推进农村转移人口市民化作为首要任务，放开县城和集镇户籍限制，有序放开中心城市落户限制，做好农村居民进城落户工作，年内转移8万人。将进城落户农民纳入城市社会保障和公共服务体系，整合城乡居民基本养老保险、基本医疗保险制度，实行农民工与城镇居民同等的保障房分配制度，将农民工子女纳入义务教育经费保障和普惠性学前教育、免费中等职业教育范围。突出规划的刚性和引领作用，把集约节约用地贯穿城镇化全过程。创新城市建设投融资体制，鼓励社会资本参与服务设施建设和运营。创新城市建设管理机制，提升城市精细化管理水平。加强住宅小区综合治理和物业市场化管理。坚持标本兼治，综合治理，严肃查处各类违规违法建设和用地行为，优化城镇建设环境。

(五)以汉江水质保护为重点，加强生态环境建设

围绕建设美丽安康，把生态文明贯穿经济社会发展全过程，扎实推进国家主体功能区建设试点示范。以高度的政治责任感保护好汉江母亲河，夯实责任，严格监管，坚持“谁审批、谁负责，谁污染、谁治理”，建立汉江水质保护应急体系和长效机制，严厉打击各类环境违法行为，确保南水北调中线工程一泓清水永续北上。加强饮用水源地保护，实施任河、旬河、岚河、月河、恒河、饶峰河等汉江支流水质监测。提高污水、垃圾无害化处理能力，确保已建成的“两厂”正常运行，加强沿江河集镇和社区污水垃圾处理设施建设，城市污水集中处理率和生活垃圾无害化处理率分别达到87%和85%以上。加强工业点源和农村面源污染防治。对高耗能、高污染企业限期整改，限期不达标的坚决予以关停。加大汉江流域水土保持、小流域治理、水污染防治、天保和退耕还林等项目实施力度；加快森林城市创建和山地森林化步伐，开展园林城市、绿化模范县、绿化示范镇和绿色家园创建活动。突出城镇(社区)周边、江河两岸、公路沿线和重要景区，绿化造林50万亩，治理水土流失面积850平方公里。加强水电站生态环境监管，做好水产养殖污染防治工作。县为主体、镇村联动，推进农村环境连片整治。开展土壤污染治理与修复试点，加强地下水资源管理。强力推进节能减排，推广应用节能新技术、新工艺、新设备，全面加强工业、建筑、交通和公共机构节能。按照完善市级、加强县级、探索镇级的要求，加强生态环保能力建设。加大环保宣传力度，倡导绿色生活，形成生态环保的生活观念、生产方式和消费模式，建立覆盖全社会的环境保护综合体系。

(六)深化改革扩大开放，增强发展动力与活力

以更大的决心和担当，把中省明确的改革部署坚决贯彻好、把市县的改革任务先行先试好、把良好的改革氛围切实维护好，全面扩大对外开放，增强发展的内生动力与活力。

推进重点领域改革。一是以转变职能为重点，创新行政管理体制，推进简政放权，接好上级下放的行政审批事项，分批取消下放市级行政审批事项。实施扩权强镇试点，稳步推进镇村、事业单位分类改革。二是围绕激发市场活力，推进工商注册制度便利化，支持民营经济转型升级，促进非公有制经济大发展。三是稳妥推进农村土地管理制度改革，研究制定农村宅基地管理办法，抓好征地制度改革试点，推进农村承包地、宅基地、集体建设用地确权登记发证工作，探索建立农村产权流转交易市场。完善农村公路养护管理机制。四是创新财政金融管理。县级以上部门全部公开预决算和“三公”经费，争取民间资本设立中小型金融机构，组建4家农村商业银行，力争金融机构贷款增长25%以上，进一步提高政府融资平台市场化融资能力。五是创新实践，完善机制，加快国家主体功能区试点示范和“飞地经济”发展，推进石泉省级城乡一体化试验示范县建设。六是围绕增强自主创新能力，建立健全以企业为主体、市场为导向、产学研用相结合的创新体系，增强科技创新在发展中的支撑和引领作用。七是围绕基本公共服务均等化，深化教育、文化、医疗卫生、社会保障、创业就业、社会治理等重点改革，推进城市公交、环卫、供水、供电、供气、垃圾处理、排污费征收等公共服务管理改革。改革经济重点工作目标责任制考核评价办法，优化指标体系。

增强招商引资实效。发挥比较优势，突出发展重点，坚持园区承载，策划一批重大项目，重点抓好装备制造、新型材料、生物医药、电子信息、现代物流和劳动密集型企业招商，围绕循环产业链条、提升产业层次，紧盯行业领军企业开展招商，增强针对性和实效性。提高房地产、矿产开发准入门槛。在加强沿海发达地区招商的同时，抓住省上建设丝绸之路经济带新起点的机遇，找准定位，及时跟进，在中省向西开放战略中有所作为。创新招商方式，强化产业和以商招商，选商选资、招大引强，抓好项目推介和跟踪落实，全年招商引资到位资金280亿元。深化与中国农科院、中国药科大学、陕西师范大学以及天津、北京石景山、江苏常州等对口协作。大力扶持培育进出口企业，提高经济外向度。

(七)加大改善民生力度，让群众得到更多实惠

顺应人民群众期待，持续加大民生投入，认真办好惠民实事，努力增加群众的幸福指数。

促进创业就业。加快发展就业主导型产业，加大劳动密集型产业引进培育力度，扶持发展中小微企业，多渠道开发就业岗位。加快公共就业服务平台建设，加强职业培训，完善创业就业服务体系，激励全民创业，激发民间创业热情，以创业带就业。建立健全劳动关系协调协商和保障农民工工资支付的长效机制，维护劳动者合法权益，构建和谐劳动关系。

实施精准扶贫。坚持分类指导，加强统筹，构建政府、市场、社会协同推进的大扶贫开发工作格局，抓紧制定扶贫项目资金到村入户实施办法、以奖代补等竞争性分配办法和扶贫项目资金管理办法；带着感情和责任，大处着眼、小处着手，加大教育、产业扶贫力度，抓好连片开发整村推进和新农村示范村建设，完成财政专项扶贫投入20亿元，实现9.45万人脱贫目标。

筑牢社会保障底线。提高城乡社会保障覆盖面和保障能力，增强托底政策的有效性，逐步建立兼顾各类人员保障待遇调整机制，完善社会保险关系转移接续政策，推进社会保障由制度全覆盖向人群全覆盖、由补缺型向适度普惠型转变。推进城乡最低社会保障制度统筹发展，加强社会救助工作，建立以最低生活保障为核心，以五保供养和医疗、住房、教育、司法救助为辅助，以临时救助、社会救助为补充的新型社会救助体系。注重把惠民政策向社会底层和弱势群体倾斜，更加关注社会底层和容易被“忽视”的群体。支持慈善、红十字事业发展，加强老龄服务，推进城乡公益性公墓建设，做好计划生育特殊困难家庭扶助工作，健全残疾人服务体系，加强市县儿童福利机构建设。

大力发展社会事业。坚持教育优先发展。扎实实施“双高双普”，巩固学前教育三年行动计划成果，实施义务教育学校标准化工程，鼓励高中多样化发展，加快发展职业和特殊教育，实施学前教育、农村义务教育薄弱学校改造、普通高中建设项目。巩固学前教育入园率和高中入学率，保障进城务工子女、留守儿童、残疾儿童义务教育。支持民办教育规范化、可持续发展，支持安康学院、安康职业技术学院实施重点学科建设。增强医疗卫生服务能力，深化医疗卫生体制改革，进一步完善新农合、城镇居民医保、医疗救助、大病保障等制度，推广医疗纠纷“第三方”调解机制以及汉滨和岚皋新农合支付、白河公共卫生服务管理、石泉新农合“就医卡”等做法。鼓励民间资本兴办医院、门诊部、诊所等医疗机构和参与公立医院改制重组，加大力度引进和培养基层医疗卫生技术人才。坚持计划生育基本国策，促进出生人口性别平衡和优生优育，提高出生人口素质。丰富群众精神文化生活，实施全国公共文化服务体系示范项目和县区文化中心、重点镇文体中心、有线电视户户通工程；支持文学艺术精品创作，推进振兴汉剧工程，广泛开展群众性文化和全民健身活动。

集中办好十件实事。一是促进就业。突出高校毕业生、城镇困难人员、复转退伍军人、农村转移劳动力和城镇“零就业”家庭五个重点，加强就业服务，免费技能培训2.3万人，新增城镇就业1.5万人，城镇登记失业率控制在4.4%以内，农村劳动力转移就业60万人。二是提升城区办学能力。市区联动、部门帮扶，推进“五校北迁”，安康职业技术学院一期工程建成；实施汉滨初中和分校、江南小学迁建，安中、汉滨高中、兴安初中、阳光学校改扩建，开工东坝、江北、黄沟小学。三是十大市政工程。实施高客站、城东大桥及一桥加固、汉调二黄广场二期、汉水龙舟文化园、江滩公园、安运司搬迁、东坝防洪、静宁市场、南环东路和重点地段交通组织(一桥南头、东堤头与中医院什字、安康大道与高速路引线交叉口)。四是 “气化安康”。加快城市天然气基础管网和设施建设，重点改造老城区居民小区管网，推进管网沿线集镇、社区天然气入户，提高天然气入户率，中心城区和县城50%群众用上天然气。五是改善环境质量。把“双创”作为推进城镇化建设的有力抓手，实现国卫创建目标。抓住“压煤、抑尘、控车、禁燃”等环节，强化大气污染防治，推进“煤改气”、“煤改电”和公交、出租车“油改气”，强化工地扬尘监管，加大机动车尾气污染治理力度，加速淘汰黄标车，未雨绸缪、治污防霾、保卫蓝天。开展村庄人居环境整治，加强美丽乡村示范点建设。创建2个省级生态县和70个市级生态镇村。六是建设文体设施。建成安康博物馆、图书馆、西城阁、高新儿童乐园，开工建设市新体育场馆、“藏一角”博物馆，加快汉江大剧院、青少年宫、科技馆、汉调二黄研究院规划建设。加强公共场所乒乓球、羽毛球、篮球、门球、网球等体育场地和设施建设，学校在保证校园安全的前提下创造条件向公众开放体育设施,新建271个镇村社区农民体育健身工程。七是提升医疗服务能力。实施市中心血站、疾控中心、安康疗养院等医疗机构北迁建设，建成市儿童医院、市妇幼保健院、精神病专科医院，新建或改扩建县级医疗服务机构5个、乡镇卫生院52个、村卫生室300所。强化社区卫生服务能力建设。八是实施安居工程。努力增加保障性住房的有效供给，完善分配与管理机制，坚决做到分配公开公平公正，切实提高入住率，使最困难群众优先获得住房保障。开工建设保障性安居工程3万套，年内基本建成2.35万套。实施农村危房改造3.5万户。建设避灾扶贫搬迁集中安置小区120个，完成2.2万户8.36万人搬迁建房主体工程。九是加强养老服务。建设市级示范性老年公寓，每个县区建成一座公益性老年公寓，新建20个社区养老日间照料中心。农村五保户集中供养率50%以上。十是发展公共交通。完成中心城市公共交通、出租车管理经营体制改革，新增和调整公交线路11条，新增和更新公交车90辆、出租车150辆。改善公交环境，加强规范管理，使公交成为城市流动的名片、文明风景线。支持有条件的县发展城市公交，并逐步向集镇延伸，提高公交覆盖率。

(八)强化基层基础，加强和创新社会治理

完善群众权益保障机制。坚持和发展“枫桥经验”，进一步完善群众诉求表达、利益协调和矛盾调处机制；推进社会治理信息化、社会化、法治化建设，完善信访源头化解、重大事项稳定风险评估机制和以人民调解为基础的大调解体系。深入推进“六五”普法，增强全民法制观念，加强法律援助工作，维护社会公平正义。认真落实民族宗教政策，依法管理宗教事务，着力解决少数民族群众生产生活困难，促进少数民族聚集区发展。

推进社会治理创新。健全城乡基层服务网络，推行社区网格化管理。以创建全国综治优秀市为抓手，深入推进平安创建。加强社会组织管理，做好流动人口、特殊人群的服务管理工作，健全农村留守儿童、留守妇女、留守老年人关爱服务体系。加强网络等新媒体的管理服务和应用，强化网吧管理，净化网络环境。加快立体化治安防控体系建设，推进社区民警专职化，壮大专职巡防队伍和群防群治力量，加强社会治安重点整治，加大“黄赌毒”打击力度，保持对违法犯罪的高压态势，增强群众安全感。严格落实安全生产责任制，全面排查整治各类安全隐患，严防重特大安全事故发生。加强食品药品监管体系建设，以最严格的监管、最严厉的处罚、最严肃的问责，确保食品药品和企业、校园、交通、消防等安全。加强应急体系和应急平台建设，提高应急处置能力，做好防灾减灾工作，保障群众生命财产安全。

认真做好国防动员、双拥共建、国家安全、优抚安置、人民防空以及统计、移民、外事、侨务、对台、史志、档案等工作，支持工会、共青团、妇联等人民团体发挥更大作用。

三、加强政府自身建设

进一步转变政府职能，深入推进责任、法治、诚信、服务、廉洁政府建设，勇于负责、敢于担当、狠抓落实，努力建设人民满意政府。

(一)坚持依法行政。坚决执行市人大及其常委会的决议和决定，自觉接受人大法律监督、工作监督和政协民主监督，提高建议提案办理工作质量。加大简政放权力度，深化行政审批制度改革，减少审批事项和环节，推进网上审批和联合审批，提高办事效率。坚持依法科学民主决策，完善公众参与、专家咨询、调研论证、风险评估、跟踪督办、情况反馈的决策机制。严格落实政府工作规则，不折不扣地执行政策、制度和工作流程。完善政府新闻发言人制度，深化政务信息公开，推进决策、管理、服务、结果公开，让政府权力在阳光下运行。拓宽民意收集、反馈渠道，善于通过新媒体倾听群众意见，加大群众网上诉求办理力度，主动接受群众和媒体监督，始终坚持在群众立场上做事、在社会监督下工作、在法治轨道上用权。

(二)持续改进作风。各级政府班子和领导干部带头树标杆、做榜样，深入开展第二批党的群众路线教育实践活动，严格执行中央八项规定，遵守党的政治、组织、工作、财经和生活纪律，坚决整治“四风”，密切同人民群众的联系。在重点工作和重大项目推进上，现场检查、办公、解决问题，争当“主攻手”，不当“二传手”，在日常工作中让大家“向我看齐”，在急难险重面前“跟我来”、“跟我上”，雷厉风行，说干就干，不拖泥带水、推诿扯皮。进一步推进思想解放，坚持问题导向，鼓励创新，宽容失败，但绝不允许碌碌无为、停步不前，以无畏的进取精神、思变的工作状态，彻底打破束缚手脚的“坛坛罐罐”，让创新实践成为主流。健全抓落实机制，任务到基层、责任到人头、工作有标准、完成有时限。发扬“钉钉子”精神，一颗接着一颗钉、一锤接着一锤敲，凡是改革发展稳定的重点任务，凡是百姓关注的重点问题，我们都要以踏石留印、抓铁有痕的作风，强化责任、一抓到底，说了算、定了干，用实绩来检验落实的效果；对涉及群众切身利益的重大民生工程，在新闻媒体向群众公开承诺，以敢于担当的锐气、破解难题的勇气、不甘落后的志气兑现承诺、取信于民；以“功成不必在我”的胸怀，干当前事为长远计，脚踏实地，持之以恒，一项一项干、一件一件抓，努力把事干成，把好事办好。强化督查问责，严格督查，刚性问责，动真碰硬，促进落实。

(三)加强廉政建设。从政府班子、组成部门和各级领导干部抓起，纪律规定是“紧箍咒”，“高压线”已带高压电，必须政令畅通、令行禁止；严格落实“约法三章”，时刻牢记权力来自人民，始终坚持公字当头、公字为先，一定心存敬畏、手握戒尺，秉持公心、用权为民，不口是心非、表里不一，对群众负责、对组织负责、对社会负责、对自己和家庭负责。严格执行党政机关厉行节约反对浪费条例，倡导勤俭节约、简朴从政，严格资金管理，严控政府开支。强化行政效能监督和问责，加大明察暗访力度，从严惩治“事难办、效率低”的行为，净化政风行风，优化投资环境。推进市、县政务服务中心标准化建设，整合部门审批事项，推行一个窗口受理、一站式审批、一条龙服务的运行模式。健全廉政风险防控体系，强化源头治理和制度防腐，加强行政审批、项目招投标、土地出让、政府采购等重点领域的行政监察和审计监督，坚决查处各种违法违纪行为。加强公务员队伍建设，严格教育管理，见微知著、狠抓苗头、建章立制，提高行政效能和服务水平，让全社会感受到市场环境、创业条件、干部作风的明显变化。

各位代表，新的使命催人奋进，人民群众寄予厚望。我们任何时候都要牢记责任、担当和落实，任何时候都要把人民的利益高高举在头顶。让我们在中共安康市委的坚强领导下，低调务实不张扬，埋头苦干不懈怠，团结和依靠全市人民，凝心聚力，同心同德，真抓实干，为建设美丽富裕新安康而努力奋斗!
